# Determination of mitoxantrone in environmental waters: a spectrophotometric method with preconcentration by salt saturated pipette-tip micro solid phase extraction using molecularly imprinted polymer

**DOI:** 10.55730/1300-0527.3728

**Published:** 2025-04-04

**Authors:** Sayyed Hossein HASHEMI, Massoud KAYKHAII, Ahmad JAMALI KEIKHA, Mina ESMAILIUN

**Affiliations:** 1 Department of Marine Chemistry, Faculty of Marine Science, Chabahar Maritime University, Chabahar, Iran; 2 Department of Analytical Chemistry, Faculty of Natural Sciences, Comenius University in Bratislava, Bratislava, Slovak Republic; 3 Department of Mechanical Engineering, Faculty of Marine Engineering, Chabahar Maritime University, Chabahar, Iran

**Keywords:** Mitoxantrane, salt saturated pipette-tip micro solid phase extraction, molecularly imprinted polymer, response surface methodology, environmental water analysis

## Abstract

In this research, a fast and simple spectrophotometric method is presented for mitoxantrone determination in various aqueous samples. It utilizes preconcentration by salt saturated pipette-tip micro solid phase extraction with a selective molecularly imprinted polymer adsorbent packed in a micropipette tip. Prior to sample loading, they were saturated with a mixture of NaCl, Mg (NO_3_)_2_.6H_2_O and KNO_3_ salts, enhancing extraction efficiency by 27.5% compared to salt-free conditions. Key microextraction parameters (amount of molecularly imprinted polymer, sample pH, eluent type/volume, and adsorption/desorption cycles) were optimized using response surface methodology and one-variable-at-a-time methods. The method achieves a low detection limit (0.2 μg L^−1^) and a wide linear range (1–1000 μg L^−1^). An enrichment factor of 49 is obtained with excellent accuracy (relative standard deviation <4.4%). The method was validated by comparing it to a standard high performance liquid chromatography protocol, as well as spiking real samples in three concentration levels. The method successfully detected low levels of mitoxantrone in various water samples.

## 1. Introduction

Mitoxantrone (MTX), an anthracycline drug used for leukemia and breast cancer treatment, poses environmental concerns due to its persistence and potential effects on aquatic life. Despite lower cytotoxicity compared to some other cancer drugs, MTX dosage increases its toxicity significantly [1–3]. MTX can enter water sources from various pathways, including excretion from drug manufacturing companies, hospitals, discard of the MTX pills into the wastewater, and pharmaceutical waste. Moreover, because of low metabolism and degradation, allergic reactions, and antibiotic resistance of the drug, 6%–11% of it can be excreted through the urine of patients taking MTX, 25% with faces and the rest in metabolized form into the sewage works through urban effluents [4–7]. Conventional water treatment methods may not eliminate MTX entirely, raising public health concerns, including allergic reactions and antibiotic resistance if it can enter water [4,5]. Toxicity response of MTX varied by exposure period and may increase exponentially by exposure time of 24 to 96 h, being its lethal concentration 50 (LC50) on Daphnia magna juveniles at 96-h (0.7 ng μL^−1^) 29-fold more toxic than at 24 h (20.3 ng μL^−1^) [6]. Amounts of 300–2000 μg L^−1^ and 300–2000 μg L^−1^ of this drug was found in human plasma and urine of patients, respectively [8,9].

Therefore, developing fast, simple, and sensitive methods for MTX detection in water samples is crucial. The method should be able to detect trace amounts of this drug in complex matrices, especially in environmental water samples, as well as in cancer treatment and pharmaceutical science.

Several analytical techniques have been established for MTX determination in drug [1], plasma and aqueous samples including extraction with molecularly imprinted polymer (MIP) and cyclic voltammetry [1], nonionic molecular interaction in surfactant micelles extraction with spectrophotometric detection [3], extraction with super paramagnetic nanoparticles and HPLC analysis [10,11], chemiluminescence [12], surface-enhanced response Raman scattering [13], and fluorescent by the glutathione stabilized gold nanoclusters [14]. Particularly, methods based on liquid chromatography / mass spectrometry (with detection of MTX of 0.7 to 20.3 ng μL^−1^) [6] and spectrophotometry (concentration in the range of 1.0 × 10^−6^ to 1.2 × 10^−5^) [15] were well developed. However, these methods often involve complex procedures, expensive instrumentation, or limitations in sensitivity. On the other hand, due to the complexity of the sample media and trace amount of MTX, an enrichment step is necessary.

Pipette-tip micro solid phase extraction (PT-μSPE) is a miniaturized and solvent-efficient preconcentration technique ideal for environmental analysis. It utilizes a tiny amount of sorbent material packed within a pipette tip. The same steps of SPE (conditioning, sample loading, elution) of traditional solid-phase extraction (SPE) but with significantly lower solvent consumption and sample volume requirements. This method also offers easy automation capabilities [16,17].

Preparation of extraction media based on MIP is a well-known and powerful strategy for selective capture of target analyte molecules in the cavities of a polymer which is capable to selectively sense and separate a special analyte. MIPs possess high affinity and specific recognition sites for the target molecule, leading to excellent reusability and stability across a wide pH range [16,17]. As a sorbent, MIP has been applied for the extraction and preconcentration of trypsin from polymeric products [18], gentamicin from water [19], aromatic amines from foodstuff [20], chlorpyrifos from fruits and fish [16,21], and profenofos from seawater samples [17].

There are reports in which MIP-SPE can be further enhanced by a technique called the salting-out effect. In almost all of them, the incorporation of salt-saturation of the sample, enhanced the selectivity and affinity of MIP toward the analytes [16,17]. The concentrated salt solution reduces the target analytes’ solubility in water, promoting its interaction with the MIP cavities and improving extraction efficiency [22,23]. In traditional extraction, usually small masses and only one kind of inorganic salt are applied, but in more recent applications, further masses of a mixture of salt are employed, that causes more efficiency because of spatial obstruction (more interaction) and an even greater reduction in the solubility of the analytes.

The goal of this paper was to evaluate the effect of presence of a high salt concentration in the samples, specifically a mixture containing NaCl, Mg(NO_3_)_2_·6H_2_O, and KNO_3_, in the samples subjected to PT-μSPE. MTX was selected as a model analyte for pipette tip micro solid phase extraction using MIP as sorbent. The main goal of this technique, named as salt saturated micro solid phase extraction by MIP (SS-MIP PT-μSPE) was application of an MIP as an adsorbent for selective microextraction of MTX in samples saturated with a mixture of common salts. To the best of our knowledge, no previous research has been found where samples were saturated with salts prior to analyte extraction using pipette-tip micro-solid phase extraction. This research presents the first application of this method for Mitoxantrone extraction.

## 2. Results and discussion

### 2.1. Characterization of MIP and NIP

**Figure 1:**
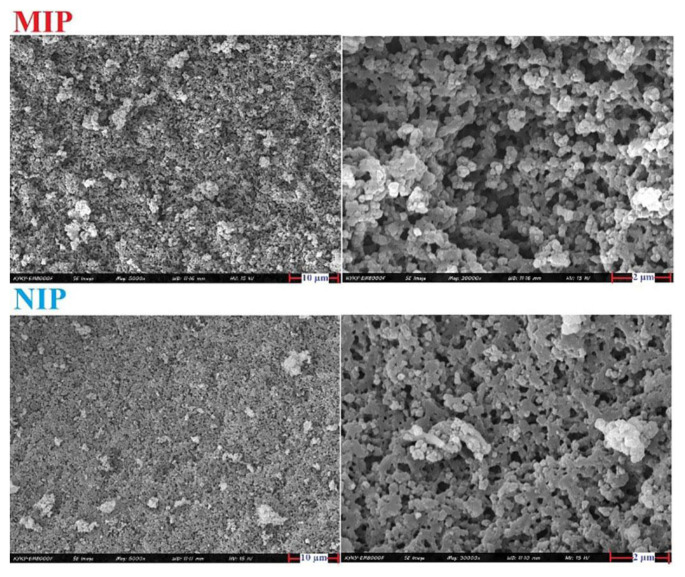
Field emission scanning electron microscopy images of MIP and NIP.

The surface morphologies of the synthesized MIP and NIP were investigated using field emission scanning electron microscopy. Images in Figure 1 reveal that both adsorbents have irregular shapes; however, the MIP appears more homogeneous and denser with more cavities and a higher surface area that facilitates adsorption of MTX. Also, this high roughness can facilitate MTX extraction. Fourier Transform Infrared (FTIR) spectra of nonimprinted polymer (NIP), leached, and unleached MIP were recorded (Figure S1). To confirm the absence of MTX on the surface of leached MIP, FTIR spectra of NIP and leached MIP is compared. The similarity of their spectra confirms this goal. For example, main peaks at 1452 cm^−1^, 1320 cm^−1^, and 1388 cm^−1^ correspond to the symmetric C-O-C stretches of monomers, 1296 cm^−1^, 1147 cm^−1^, and 2986 cm^−1^ are related to OH stretching of MAA that are almost the same as in NIP. The absence of the peaks of C=C at around 1585 cm^−1^ indicates that the polymer is properly formed due to double bands are incorporated into the polymer framework. The FTIR spectra of the MIP indicates a group of bands assigned to C-H (2953 cm^−1^), C=O (1723 cm^−1^), and C-O (1253 cm^−1^), that are similar in the NIP spectra, proving the similarity of backbone structures. These observations proved that all MTX was leached without affecting the main structure of the MIP. The peak at around 1240 cm^−1^ and 470 cm^−1^ in unleached MIP related to template molecule that leached MIP was removed.

### 2.2. Experimental condition optimization

To achieve obtain high MTX recovery, the parameters affecting SS-MIP-PT-μSPE μSPE method were investigated and investigated using a combination of one-variable-at-a-time optimization and response surface methodology (RSM). MIP preparation conditions, sample solution pH, sorbent mass, eluent volume, and elution cycles were optimized using two different techniques: central composite design (CCD) and Box-Behnken design (BBD). The number of adsorption cycles and type of desorption solvent were studied by one-variable-at-a-time method. For optimization runs, a standard solution of MTX with a concentration of 400 μg L^−1^ was employed. Each experiment was repeated at least three times, and the average was used as the data point.

#### 2.2.1. Type of elution solvent

Eluent selection is crucial for SPE as it should efficiently elute the target analyte from the sorbent while minimizing solvent volume. Various eluents, including pure and mixed solvents like acetonitrile, methanol, acetic acid, ethanol, acetone, and HCl (0.5 and 1.0 mol L^−1^), were tested. Among them, a 1:1 mixture of acetone and acetic acid exhibited the best elution efficiency due to its suitable polarity and high dielectric constant. This disrupts the interaction between MTX and the MIP cavities, promoting its release. Mitoxantrone, with its polar functional groups, is most effectively eluted using polar solvents. The analyte can form hydrogen bonds with the solvent mixture of acetone and acetic acid in a 1:1 ratio [24,25]. Consequently, the acetone:acetic acid (1:1) mixture was chosen as the optimal eluent for further experiments.

#### 2.2.2. Number of extraction cycles

The technique of aspirating a sample into the conditioned pipette tip and dispensing it back into the sample vial is termed an “aspiration/dispensing cycle”. The number of extractions (number of aspiration/dispensing cycles) is a critical factor in PT-μSPE, as multiple passes are often necessary to achieve equilibrium between the adsorbent and the sample solution. However, unnecessary repetitions should be avoided to minimize extraction time. With a lower number of extractions, the adsorption of MTX is incomplete, while with a higher number, the target molecule can desorb back into the sample solution and MIP [26]. To determine the optimal number of MTX loading cycles, the number of aspiration/dispensing cycles was optimized. Extraction cycles between 2 and 6 were investigated, and it was found that after 4 cycles, optimal extraction was achieved. Consequently, 4 cycles were employed for subsequent experiments.

#### 2.2.3. Response surface methodology

RSM is a powerful statistical technique that utilizes strategically designed experiments to optimize multiple factors simultaneously and identify their interactions affecting a desired response [16,17]. In this study, RSM was employed to optimize some of the parameters influencing the performance of SS-MIP-PT-μSPE for MTX extraction. Two common RSM designs, CCD and BBD, were evaluated to select the most suitable approach. The optimized factors investigated were: pH (A or X_1_), mass of sorbent (B or X_2_), volume of eluent (C or X_3_), and number of elution cycles (D or X_4_). The pH of the sample is a significant parameter in preconcentration of MTX on MIP because of keeping the neutral state of the molecule. Eluent volume is considered as another significant variable which improved the response of MTX. The protocol of aspirating of standard solution into the conditioned pipette tip and dispensed back into the solution vial is named “an absorption cycle”. Number of absorption cycles is also an important variable for enrichment in the μSPE. The design of the experiment is indicated in supplementary data, Tables S1 and S2.

In a function consist of A, B, C and D, the relationship of the analytical instrument on the variables can be obtained utilizing quadratic polynomial equation (eq. 1):


eq. 1 $$\begin{array}{l} \;\;\;\text{Y }(\text{predicted response})=\beta_{0}\;(\text{constant})+\Sigma \beta_{\text{i}}\;(\text{linear effect})\;{\text{X}}_{\text{i}}\;(\text{coded independent variable})+\Sigma \beta_{\text{ii}}(\text{quadratic response})\\ {\text{X}}_{\text{ii}}\;(\text{coded independent variable})+\Sigma \beta_{\text{ij}}(\text{coefficient of the interaction factor})\;{\text{X}}_{\text{i}}{\text{X}}_{\text{j}}\;(\text{coded independent variables})+\text{e}\\ \left(\text{random error}\right) \end{array}$$

In equation 1 and 3, the relationship of the signal and 4 variables are excessed:


eq. 2          Y (for CCD)=(-15.65476+(2.96154×A))+(3.05035×B)+(0.026008×C)+(0.18596×D)-(0.014784×A×B)-(1.30550×10-4×A×C)+(1.59734×10-3×A×D)-(5.34922×10-5×B×C)+(1.32788×10-3×B×D)-(1.03042×10-7×C×D)-(0.23667×A2)-(0.48865×B2)-(6.25047×10-5×C2)-(0.032254 D2))^(1/1.5)

eq. 3 $$\begin{array}{l} \;\;\;\text{Y }(\text{for BBD})=-10.88802+(1.65750\times \text{A})+(3.08500\times \text{B})+(0.019733\times \text{C})+(0.13229\times \text{D})-(5.0\times 10^{-3}\times \text{A}\times \text{B})+\\ (5.0\times 10^{-4}\times \text{A}\times \text{C})-(5.0\times 10^{-3}\times \text{A}\times \text{D})-(1.2\times 10^{-3}\times \text{B}\times \text{C})-(0.14417\times {\text{A}}^{2})-(0.46667\times {\text{B}}^{2})-(4.71667\times 10^{-5}\times \\ {\text{D}}^{2})-(0.016354\times {\text{D}}^{2}) \end{array}$$

By solving the equations of *∂*Y/*∂*A = 0, *∂*Y/*∂*B = 0, *∂*Y/*∂*C = 0, and *∂*Y/*∂*D = 0, the optimal variables can be obtained [16,17]. In Tables S3 and S4, the analysis of variance (ANOVA) are performed. The amount for optimum variables are: pH (A) = 5.99 (for BBD) and 6.12 (for CCD), mass of sorbent (B) = 3.0 mg (for BBD and CCD), volume of eluent (C) = 203.0 μL (for BBD) and 200.0 μL (for CCD) and number of elution cycles (D) = 3.1 (for BBD). Reasons for optimal number of variables can be expressed as below.

The decrease in absorption at lower or higher pH could be related to the hydrolysis (or conversion of MTX to an ionic form that cannot be effectively adsorbed on MIP) of MTX in strongly acidic or alkaline samples. Due to the presence of ionizable functional groups in the analyte, sample pH can affect the extraction efficiency of the target molecule. Ideally, the analyte should be in its deionized form to be efficiently extracted by the adsorbent via hydrogen bonding interactions. Thus, protonation and deprotonation of ionizable functional groups on the adsorbent surface can be influenced by solution pH, affecting the extraction efficiency of the compound [25]. Increasing the amount of sorbent increases the surface area available for MTX adsorption [27]. Maximum response was obtained with 3.0 mg of MIP. Further increases in sorbent loading decreased the signal and prolonged sample passage time [26].

When the volume of eluent is more than optimal volume, a decrease in response observed is happened because of the dilution of it [25]. In a low volume of eluent, the enrichment of MTX cannot be obtained efficiently [27]. In low number of elution passage, the elution of MTX from the MIP is incomplete and in higher runs, the target can go back to the MIP [26]. F-value of 28.47 (for BBD) and 287.48 (for CCD) explains the model is excellent. The regression model for the compound excessed in determination coefficient (R^2^ = 0.9962 (for CCD) and 0.9637 (for BBD), showing which 0.38 % (for CCD) and 3.63 % (for BBD) of the variation cannot be excessed with CCD and BBD. The adjusted determination coefficient at adjusted R^2^ = 0.9928 (for CCD) and 0.9299 (for BBD) shown which the CCD and BBD were suitable. The prediction R^2^ of 0.9861 (for CCD) and 0.8229 (for BBD) were showed high predictive power of the BBD and CCD. The amounts in Table S3 and S4 shown which A, A^2^, B^2^, C^2^, D^2^ (for CCD) and BC, A^2^, B^2^, C^2^, D^2^ (for BBD) are effective terms. The F-value of lack of fit of 1.51 (for CCD) and 1.97 (for BBD) explained which they are not significant relative to the pure error. The observations exceeded, which CCD has better application, and it is applied in further research. Figure S2 shows a two-dimensional response surface in versus two factors at the middle amount of the two variables.

### 2.3. Analytical performance

#### 2.3.1. Method evaluation

Analytical features of the PT-μSPE were studied by plotting an external calibration curve. The technique proved to be linear (R^2^ = 0.9955) over the amount range of 1.0 to 1000.0 μg L^−1^ of MTX, by the equation of A = 2.1087C (mg L^−1^)+0.1018, where C is the concentration of the analyte in mg L^−1^. The limit of detection (LOD) and limit of quantification (LOQ) were achieved according to 3S_d_ and 10S_d_ criteria (S_d_ is the standard deviation of 10 repeated determination of the blank) and obtained to be 0.2 and 0.7 μg L^−1^, respectively. The enrichment factor (EF) of the method was calculated as the ratio of calibration curve slopes after SS-MIP-PT-μSPE extraction and without doing extraction which was achieved as 137-fold. The intra-day precision of suggested PT-μSPE investigated as RSD was ranged between 1.7% and 4.7%, and the interday reproducibility was better than 4.2% in all cases. The repeatability of proposed protocol, expressed as RSD was 3.2 that was evaluated with carrying out eight replicates of the spiked sample in the 75 μg L^−1^ concentration.

Recovery (R%) for all samples was achieved based on the eq. 4. Samples were spiked with an explained volume of a standard of MTX.


eq. 4 $$\text{R}\%=({\text{C}}_{\text{found}}-{\text{C}}_{\text{real}})/{\text{C}}_{\text{added}}$$

Where C_found_, C_real_ and C_added_ are MTX amount determined after analysis of 10.0 mL spiked sample, the concentration of MTX determined prior spiking, and concentration of standard solution applied for spiking of real sample, respectively.

A comparison of PT-μSPE with previously reported methods for the extraction and detection of MTX in various real samples is presented in Table 1. As shown, PT-μSPE/HPLC-UV with a deep eutectic solvent-doped graphite as the adsorbent demonstrates higher RSD and lower precision compared to SS-MIP-PT-μSPE. When compared to the MIP sensor coupled with cyclic voltammetry, SS-MIP-PT-μSPE exhibits a broader linear range (LR). Additionally, it consumes less solvent, sample, and eluent compared to methods such as the MIP sensor coupled with cyclic voltammetry, HPLC-UV, and the association complex combining MTX with eosin Y reagent, which aligns with the principles of green analytical chemistry. However, the method’s higher LOD compared to techniques such as MIP sensor coupled with cyclic voltammetry and HPLC-FL is attributed to the higher sensitivity of the latter instruments. Another drawback of the proposed protocol is its higher sorbent consumption compared to PT-μSPE using deep eutectic solvent-doped graphite as the sorbent. Furthermore, the shorter LR of PT-μSPE compared to technique number 3 in Table 1 is due to the higher sensitivity of fluorescence detectors. On the other hand, the enrichment factor (EF) of the proposed method (137-fold) is superior to that of the deep eutectic solvent-doped graphite (80-fold), highlighting another advantage of the technique.

#### 2.3.2. Reusability and stability of prepared SS-MIP-PT-μSPE

The stability and reusability of the synthesized MIP were evaluated by analyzing a standard solution of MTX at a concentration of 250 μg L^−1^ five times using the same sorbent. After each extraction-elution cycle, the MIP was washed multiple times with a mixture of acetone and acetic acid (1:1) to ensure that no target molecule remained on it. There was a very slight decrease in extraction efficiency after five adsorption-elution cycles, with the extraction efficiency reduced by 5.6%. This observation demonstrated that the MIP possessed enough stability and reusability. The reduction in extraction efficiency might be attributed to damage to some of the imprinted cavities in the sorbent. In contrast, the NIP showed no significant change in extraction efficiency after repeated extractions, as it lacked imprinted cavities.

#### 2.3.3. Evaluation of sensitivity and imprinting factor of MIP

**Figure 2:**
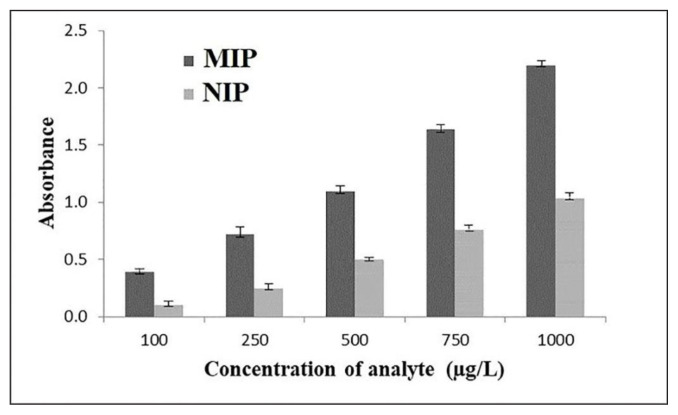
Comparison of MIP and NIP sorbent.

The sensitivity of MIP compared to NIP for the enrichment of MTX was investigated. An extraction was carried out on a range of the amount of standard solutions of MTX (100, 250, 500, 750, and 1000 μg L^−1^) in the same factors by both MIP and NIP. On average, MIP indicated 64% more adsorption (due to the further porosity of MIP that makes a higher adsorptive site, and also due to the more selectivity of it toward the analyte). While NIP can only adsorb the analyte because of surface adsorptivity. Results are presented in Figure 2.

The imprinting factor (IF) was calculated to assess the recognition ability of the MIP: IF = EF_MIP_/EF_NIP._

Where EF_MIP_ is the enrichment factor of MTX extracted using MIP, and EF_NIP_ is the enrichment factor of MTX extracted using NIP under the same conditions. The calculated IF was 2.49 across the concentration range of standard MTX solutions (100, 250, 500, 750, and 1000 μg L^−1^). Additionally, the SS-MIP-PT-μSPE showed weaker extraction efficiency for levofloxacin and 6-mercaptopurine compared to NIP-PT-μSPE. These findings confirm the specific selectivity of the MIP for MTX.

#### 2.3.4. Comparison of the performance of extraction with and without saturation by salt

**Figure 3:**
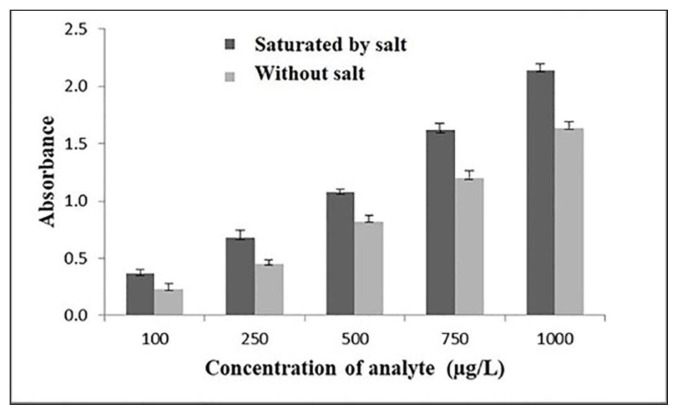
Effect of saturating by salt on the extraction efficiency.

MIP-PT-μSPE and SS-MIP-PT-μSPE were compared together in an experiment for the extraction of MTX from 5 standard mixtures by amounts of 100, 250, 500, 750, and 1000 μg L^−1^ in optimum variables. Figure 3 compares two situations of extraction in the presence and in the absence of salts. As can be seen, saturation of the solution with salts enhanced the analytical signal, which means that a higher extraction efficiency was obtained. In average, 27.5% enhancement was observed which is due to the salting-out effect. Presence of high amount of salt reduced the solubility of MTX in the aqueous phase; so, further analyte can be entered into the MIP. Using different salts which contain different ions can even intensify the salting-out effect due to the more ions which are presented in the solution.

#### 2.3.5. Analysis of real samples

**Figure 4:**
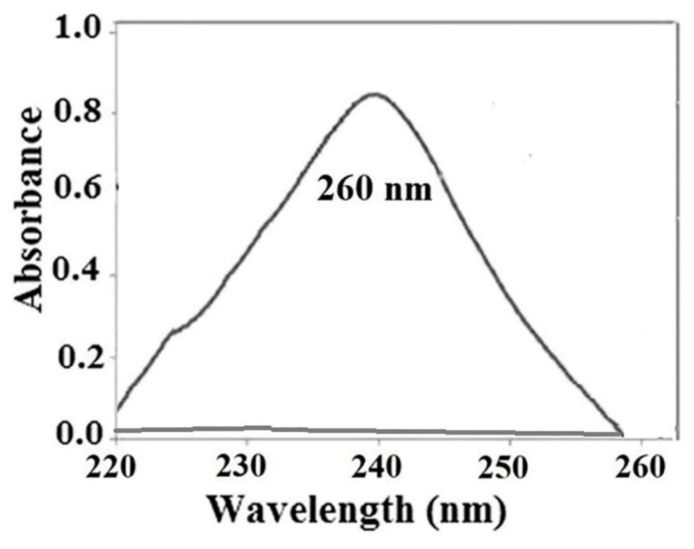
The absorbance spectrum of MTX against blank solution after SS-MIP-PT-μSPE of a seawater sample spiked with 400 μg L^−1^ of the analyte.

To confirm the performance of the SS-MIP-PT-μSPE technique for real sample analysis, samples of wastewater, seawater, and tap water were analyzed by this method. Moreover, each sample was spiked by MTX in three various amounts of concentrations to evaluate the effect of the media. As an example, Figure 4 shows the absorbance spectrum of MTX of a spiked seawater sample spiked by 400 μg L^−1^ of the drug against a blank solution after SS-MIP-PT-μSPE. The results are explained in Table 2.

#### 2.3.6. Accuracy of the method

To study the validity of the developed method, detection of an actual media spiked with 10 mL of 20 μg L^−1^ standard solutions of MTX was performed using SS-MIP-PT-μSPE and compared using a standard technique of HPLC (sec. section of “3.6”). A Student’s t-test at 95% confidence limit showed that statistically there is no significant difference between the observations.

#### 2.3.7. Effect of the presence of interferences

Levofloxacin and 6-mercaptopurine as interference agents generally existed with MTX in actual media. To investigate the effect of them, a 10 mL mixture of 400 μg L^−1^ of MTX was formed, consisting of a similar amount of the interfering analytes and determined using the PT-μSPE. No interferences were observed because of the high selectivity of MIP.

#### 2.3.8. Robustness

The suggested spectrophotometric system was validated for robustness by testing its performance under slight variations in system parameters, including pH (6.0 ± 0.2), sorbent mass (3.0 ± 0.2 mg), and eluent volume (200 ± 20 μL). The robustness was assessed by measuring percent recovery and standard deviation values. The slight variations in these parameters were found to have no significant impact on absorbance (instrument response), demonstrating the robustness of the proposed SS-MIP-PT-μSPE coupled spectrophotometry.

#### 2.3.9. Physicochemical properties

A sensor is a device that detects or measures a physical property and records, indicates, or otherwise responds to it. For a chemical sensor, the “physical property” refers to the presence of the target analyte. Critical factors in the application of MIPs in sensors include the imprinting factor (IF), binding capacity (BC), and response time (RT). The binding capacity is defined as the ratio of the concentration of the template analyte adsorbed from the test solution to its initial concentration, expressed as a percentage. The response time refers to the duration required to achieve 63.2% of the final signal after the application of the stimulus. The imprinting factor (IF) is discussed in Section 2.3.3, “Evaluation of Sensitivity and Imprinting Factor of MIP”. To evaluate the total capacity of the SS-MIP-PT-μSPE for MTX, extractions were carried out under optimal conditions using varying concentrations of the target analyte in 10 mL of solution. The adsorptive capacity of the MIP was determined to be 250 μg for MTX.

### 2.4. Conclusions

In this paper, a molecularly imprinted polymer adsorbent was synthesized and used for pipette-tip micro solid phase extraction of mitoxantrone followed by its spectrophotometric determination in water, seawater, tap water and wastewater. Before extraction, samples were saturated with three inorganic salts which caused 27.5% higher extraction efficiency with a shorter extraction time, due to salting-out effect. The results indicated that the suggested method has advantages such as being simple, economical and fast, and could potentially be used as an alternative method for the enrichment of low concentration of mitoxantrone in complex matrices. For spiked water/seawater samples, mitoxantrone at the amount of as trace as 9.7 μg L^−1^ could be detected, and precision was better than 3.7% (as relative standard deviation (%)). The reproducibility and repeatability of the method, expressed as RSD, were better than 4.7% and 3.2%, respectively. The stability and reusability of the synthesized MIP were evaluated by analyzing a standard solution of MTX at a concentration of 250 μg L^−1^ over five consecutive uses. The use of MIP offers several advantages, including high stability under various conditions, ease of preparation, low cost, and specific recognition of target analytes.

## 3. Experimental

### 3.1. Materials

Mitoxantrone (purity 99.3%) bought of Sigma-Aldrich Co. (St. Louis, MO, USA). All other chemical used were of analytical grade and supplied by Merck Company (Darmstadt, Germany). A 100 mg L^−1^ stock solution of MTX was achieved with mixing exactly 10 mg of MTX in 100 mL distilled water. Standard solutions were made daily by proper dilution of the mother solution in ultra-pure water. For the adjustment of pH of the solution, either 1 M HCl or NaOH was used.

### 3.2. Apparatus

A Steroglass model 2014 spectrophotometer (Italy) was utilized for the absorption measurements. For spectrophotometric measurement, a wavelength of 260 nm was used. pH of the samples was determined with a Metrohm pH meter (Switzerland, model 630). Structure elucidation of MIP was evaluated by a Fourier transform infrared spectroscopy (FTIR) instrument made by Perkin-Elmer company (Bucks, UK). To characterize the morphology of polymers, a field emission scanning electron microscope (Sigma VP, Germany) by acceleration voltage of 15 KV was employed. A Knauer HPLC (Germany) combined by UV-Vis diode array detection was used to explain the accuracy of the technique.

### 3.3. Synthesis of MIP and nonimprinted polymer

**Figure 5:**
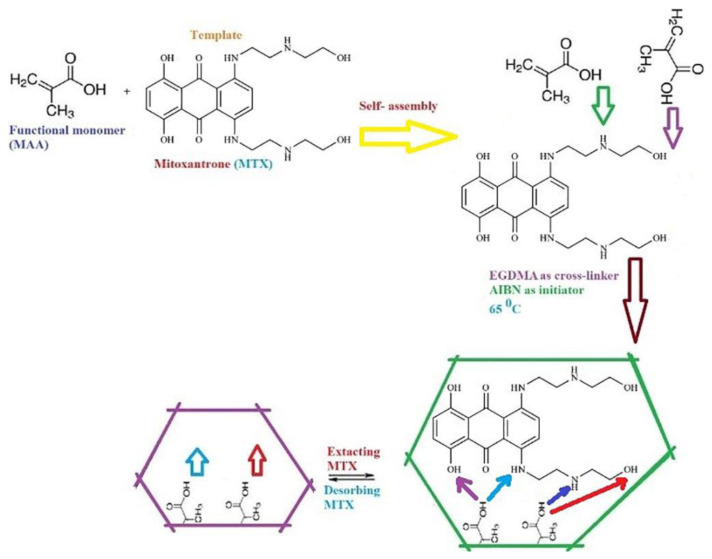
Preparation of MIP.

MIP was synthesized by the following polymerization process. 0.5 mmol (0.22 g) of MTX as template, 2 mmol (0.88 g) of methacrylic acid (MAA) as monomer, 3.8 mL of ethylene glycol dimethacrylate (EGDMA) as cross-linker, and 80 mg of 2,2′-azoisobutyronitrile (AIBN) as starter were dipped in 6.0 mL of CH_3_OH. Solution was deoxygenated by N_2_ gas for 7 min. For formation of the polymer, the vial was placed for 2 h at 65 ^°^C. The resulting solid MIP was washed 3 times by CH_3_OH, then dried in the air and ground. For delete of the remaining compound of the MIP, the polymer was washed by 0.5 mol L^−1^ HCl until no analyte remained in the eluent (tested by spectrophotometer). Finally, the MIP was washed three times with CH_3_OH and dried. Preparation of MIP is shown schematically in Figure 5. As shown in Figure 5, potential interactions between MTX and MIP sorbent can be of hydrogen band type, occurring between NH and OH groups of the analyte and OH groups of MAA monomer. The nonimprinted polymer (NIP) was prepared similar to MIP, but without MTX.

### 3.4. Real samples preparation

The applicability of the method was evaluated using complex matrices such as seawater, wastewater, and tap water to demonstrate its effectiveness in various real-world samples. While environmental water samples may have varying salt compositions, the SS-MIP-PT-μSPE method can be adapted accordingly. For example, sweaters already contain many salts, while in matrices such as tap water and wastewater, the type and concentration of the salts are vastly different. It would be of interest to see if the method can be used for different types of matrices.

Seawater samples were collected from two seawater stations in Chabahar Bay (southeast of Iran). To remove suspended material, they were centrifuged at 5000 rpm for 10 min and filtered using a 450 μm Nylon membrane filter. Wastewater samples were collected from municipal sewage and a student’s dormitory and were filtered under vacuum pressure using a glass fibre with a pore size of 11 μm, followed by 0.7 μm pore size glass fibre filters to minimize cartridge clogging during the μSPE step. Glass fibre filters were rinsed with methanol and deionized water to prevent cross-contamination.

Tap water samples were prepared similarly to seawater samples, with no additional treatment steps.

Before extraction, the pH of 10.0 mL aliquots of each sample was adjusted to the optimal pH of 6.0, and then 1.12 g NaCl, 1.50 g Mg (NO_3_)_2_.6H_2_O, and 3.35 g KNO_3_ were added to the solutions in order to be saturated by these salts. To the seawater samples, no salt was added, since they are saturated with many salts. The criteria for the choice of these salts were the lack of similar ions between them.

### 3.5. Extraction procedure

**Figure 6:**
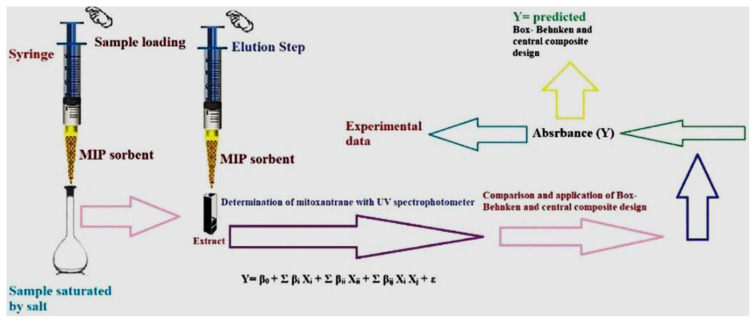
μSPE procedure.

The SS-MIP-PT-μSPE procedure is schematically presented in Figure 6. 3.0 mg of the synthesized MIP was placed in a 150 μL pipette tip and then PT was connected to a 10 mL syringe and utilized for the microextraction of MTX from the samples without any further steps. Prior to extraction, the pipette-tip was pretreated with 200 μL of methanol and water. 100 μL of a salt-saturated standard or the sample solution was added into the PT and returned back to the solution. This process was repeated 4 times. After that, the sorbent was washed by 200 μL of methanol to remove the co-adsorbed matrix materials from it. After extraction, in order to elute the analyte from the PF column, 200 μL of acetone: acetic acid (1:1) was soaked into it and sent out in a micro-cell of the spectrophotometer. This process was repeated 3.0 times. Finally, 100 μL of the eluted solution was introduced into the spectrophotometer for analysis. Absorbance determinations were performed at λ_max_ = 260 nm.

### 3.6. Analysis by reversed phase high performance liquid chromatography

To confirm the suggested method, real samples were also investigated using HPLC and were placed under the following conditions.^4^ A 100 mm Hypersil GOLD™ C_18_ Selectivity HPLC Column (Thermo Fisher Scientific Inc., USA), equipped with a C_18_ guard column was applied using isocratic elution. The mobile phase includes a (75:25 v/v) solution of triethylammonium formate buffer:acetone. Buffer was obtained with mixing of 0.2 mol of formate and 0.1 mol trimethylamine in 1L H_2_O and its pH was adjusted to 3.5 at 24 ^°^C. The detection wavelength was 260 nm.

## Supplementary materials

**Table S1:** BBD of parameter and their corresponding experimental and predicted values.

No	pH	Mass of sorbent	Volume of eluent	Elution cycles	Response	Predicted	Error%
1	5	2.5	200	3	0.63	0.66	−4.76
2	5	3	150	3	0.68	0.68	0.00
3	5	3	200	1	0.74	0.71	4.05
4	5	3	200	5	0.76	0.74	2.63
5	5	3	250	3	0.66	0.66	0.00
6	5	3.5	200	3	0.67	0.68	−1.49
7	6	2.5	150	3	0.68	0.65	4.41
8	6	2.5	200	1	0.72	0.73	−1.39
9	6	2.5	200	5	0.75	0.75	0.00
10	6	2.5	250	3	0.76	0.73	3.95
11	6	3	150	1	0.71	0.73	−2.82
12	6	3	150	5	0.73	0.74	−1.37
13	6	3	200	3	0.90	0.93	−3.33
14	6	3	200	3	0.94	0.93	1.06
15	6	3	200	3	0.96	0.93	3.12
16	6	3	200	3	0.93	0.93	0.00
17	6	3	200	3	0.92	0.93	−1.09
18	6	3	200	3	0.94	0.93	1.06
19	6	3	250	1	0.74	0.75	−1.35
20	6	3	250	5	0.76	0.77	−1.32
21	6	3.5	150	3	0.74	0.72	2.70
22	6	3.5	200	1	0.73	0.75	−2.74
23	6	3.5	200	5	0.76	0.77	−1.32
24	6	3.5	250	3	0.70	0.69	1.43
25	7	2.5	200	3	0.65	0.66	−1.54
26	7	3	150	3	0.61	0.63	−3.28
27	7	3	200	1	0.75	0.72	4.00
28	7	3	200	5	0.73	0.72	1.37
29	7	3	250	3	0.69	0.71	−2.90
30	7	3.5	200	3	0.68	0.67	1.47
	5.5	2.8	200	3	0.89	0.87	2.25
	6.5	3	220	3	0.90	0.89	1.11
	6	3.5	250	4	0.67	0.68	−1.49
	7	3.2	180	2	0.74	0.72	2.70
	6	2.5	200	1	0.75	0.73	2.67
	6	3.7	200	5	0.65	0.66	−1.54
	7.5	3	210	3	0.63	0.61	3.17
	6	3.2	260	3	0.77	0.75	2.60
	6	3	200	6	0.81	0.80	1.23

**Table S2:** CCD of parameter and their corresponding experimental and predicted values.

No	pH	Mass of sorbent	Volume of eluent	Elution cycles	Response	Predicted	Error%
1	5	2.5	150	1	0.30	0.30	0.00
2	5	2.5	150	5	0.31	0.32	−3.23
3	5	2.5	250	1	0.32	0.33	−3.13
4	5	2.5	250	5	0.34	0.34	0.00
5	5	3	200	3	0.75	0.72	4.00
6	5	3.5	150	1	0.33	0.35	−6.06
7	5	3.5	150	5	0.35	0.37	−5.71
8	5	3.5	250	1	0.37	0.36	2.70
9	5	3.5	250	5	0.39	0.39	0.00
10	6	2.5	200	3	0.84	0.84	0.00
11	6	3	150	3	0.85	0.82	3.53
12	6	3	200	1	0.83	0.83	0.00
13	6	3	200	3	0.92	0.93	−1.09
14	6	3	200	3	0.95	0.93	2.11
15	6	3	200	3	0.94	0.93	1.06
16	6	3	200	3	0.91	0.93	−2.20
17	6	3	200	3	0.93	0.93	0.00
18	6	3	200	3	0.94	0.93	1.06
19	6	3	200	5	0.86	0.85	1.16
20	6	3	250	3	0.80	0.82	−2.50
21	6	3.5	200	3	0.86	0.86	0.00
22	7	2.5	150	1	0.44	0.45	−2.27
23	7	2.5	150	5	0.47	0.47	0.00
24	7	2.5	250	1	0.46	0.44	4.35
25	7	2.5	250	5	0.48	0.47	2.08
26	7	3	200	3	0.78	0.80	−2.56
27	7	3.5	150	1	0.46	0.45	2.17
28	7	3.5	150	5	0.49	0.49	0.00
29	7	3.5	250	1	0.45	0.45	0.00
30	7	3.5	250	5	0.48	0.48	0.00
	5.5	2.8	200	3	0.87	0.85	2.30
	6.5	3	220	3	0.90	0.89	1.11
	6	3.5	250	4	0.72	0.72	0.00
	7	3.2	180	2	0.74	0.74	0.00
	6	2.5	200	1	0.70	0.73	−4.29
	6	3.7	200	5	0.66	0.68	−3.03
	7.5	3	210	3	0.60	0.58	3.33
	6	3.2	260	3	0.77	0.76	1.30
	6	3	200	6	0.73	0.73	0.00

Figure S1 FTIR spectra of MIP and NIP.

**Table S3:** ANOVA analysis for MTX response (BBD).

Source	Sum of Squares	df	Mean Square	F Value	p-value Prob > F	% PC= (SS/ Σ SS) × 100
Model	0.273045	14	0.019503	28.47185	< 0.0001	
A-pH	7.5 × 10 ^−5^	1	7.5 × 10 ^−5^	0.109489	0.7453	7.5 × 10 ^−5^
B-mass of Sorbent	0.000675	1	0.000675	0.985401	0.3366	0.18
C-volume of Eluent	0.002133	1	0.002133	3.114355	0.0980	0.57
D-Cycles of Elution	0.000833	1	0.000833	1.216545	0.2874	0.22
AB	2.5 × 10 ^−5^	1	2.5 × 10 ^−5^	0.036496	0.8511	2.5 × 10 ^−5^
AC	0.0025	1	0.0025	3.649635	0.0754	0.67
AD	0.0004	1	0.0004	0.583942	0.4566	0.11
BC	0.0036	1	0.0036	5.255474	0.0368	0.97
BD	0	1	0	0	1.0000	0
CD	0	1	0	0	1.0000	0
A^2	0.142519	1	0.142519	208.057	< 0.0001	38.45
B^2	0.093333	1	0.093333	136.253	< 0.0001	25.18
C^2	0.095344	1	0.095344	139.1884	< 0.0001	25.72
D^2	0.029344	1	0.029344	42.83803	< 0.0001	7.92
Residual	0.010275	15	0.000685			
Lack of Fit	0.008192	10	0.000819	1.966	0.2359	not significant
Pure Error	0.002083	5	0.000417			
Cor Total	0.28332	29				

PC %: Percent contribution, SS: Sum of squares

Figure S2 Two-dimensional response surface as the functions of two variables at the center level of other variables.

**Table S4:** ANOVA analysis for MTX response (CCD).

Source	Sum of Squares	df	Mean Square	F Value	p-value Prob > F	% PC= (SS/ Σ SS) × 100
Model	2.444452	14	0.174604	287.4812	< 0.0001	significant
A-pH	0.056218	1	0.056218	92.56246	< 0.0001	15.91
B-mass of Sorbent	0.002392	1	0.002392	3.938787	0.0658	0.68
C-volume of Eluent	0.000175	1	0.000175	0.288047	0.5993	0.049
D-Cycles of Elution	0.00258	1	0.00258	4.2473	0.0571	0.73
AB	0.000874	1	0.000874	1.439434	0.2488	0.25
AC	0.000682	1	0.000682	1.122459	0.3062	0.19
AD	0.000163	1	0.000163	0.268863	0.6117	0.046
BC	2.86 × 10 ^−5^	1	2.86 × 10 ^−5^	0.047113	0.8311	2.86 × 10 ^−5^
BD	2.82 × 10 ^−5^	1	2.82 × 10 ^−5^	0.046451	0.8323	2.82 × 10 ^−5^
CD	1.7 × 10 ^−5^	1	1.7 × 10 ^−9^	2.8 × 10 ^−6^	0.9987	1.7 × 10 ^−5^
A^2	0.145118	1	0.145118	238.9333	< 0.0001	41.08
B^2	0.038665	1	0.038665	63.66137	< 0.0001	10.94
C^2	0.063264	1	0.063264	104.1631	< 0.0001	17.91
D^2	0.043125	1	0.043125	71.00466	< 0.0001	12.21
Residual	0.00911	15	0.000607			
Lack of Fit	0.006844	10	0.000684	1.510326	0.3395	not significant
Pure Error	0.002266	5	0.000453			
Cor Total	2.453562	29				

PC %: Percent contribution, SS: Sum of squares

## Figures and Tables

**Table 1:** Comparison of SS-MIP-PT-μSPE with similar methods.

Sample matrix	Extraction method	Instrument	Linear range (μg L^−1^)	LOD (μg L^−1^)	Volume of the eluent (μL)	RSD (%)	Sorbent mass (mg)	EF	Ref.
Pharmaceuticals, urine	MIP	Cyclic voltammetry	0.013–4.44	0.013 M	NM	≤ 5.1	NM	NM	[1]
Plasma	NM^1^	HPLC-UV	0.0001–0.44	0.001	5000	≤ 13.7	NM	NM	[8]
Human biofluids	Association complex combining MTX and the eosin Y reagent	Spectro-fluorimetry	70–2500	16	800	≤ 1.1	NM	NM	[9]
Seawater, drug, urine and blood	Deep eutectic solvent-doped graphite PT-μSPE	Spectro-photometry	1–1000	0.2	200	≤ 4.4	1.0	80	[28]
Seawater, tap water, wastewater	SS-MIP-PT-μSPE	Spectro-photometry	1.0–1000.0	0.2	200	≤4.1	3.0	137	This work

1 NM= Not Mentioned

**Table 2:** Analysis of real samples by SS-MIP-PT-μSPE.

Sample	Analyte added (μg L^−1^)	Analyte found (μg L^−1^)	Recovery (%)	RSD% (n =3)
Seawater (taken from Chabahar Maritime University coat)	0	not detected	-	-
	10	9.7	97.0	2.1
	20	19.9	99.5	3.3
	50	49.6	99.2	3.7
Seawater (taken from Lipar, Oman sea)	0	not detected	-	-
	10	9.8	98.0	3.1
	20	19.8	99.0	3.0
	50	49.7	99.4	2.7
Tap water	0	not detected	-	-
	10	9.9	99.0	1.1
	20	19.7	98.5	1.3
	50	49.1	98.2	2.8
Wastewater	-	1.1	-	2.3
	10	11.0	99.1	3.2
	20	21.0	99.5	3.9
	50	50.2	98.2	4.1
