# Supplementary figures and images for: Determination of mitoxantrone in environmental waters: a spectrophotometric method with preconcentration by salt saturated pipette-tip micro solid phase extraction using molecularly imprinted polymer
Source: Turk J Chem. 2025 Apr 4;49(3):267–78. doi: 10.55730/1300-0527.3728 (PMC12253970; doi:10.55730/1300-0527.3728)

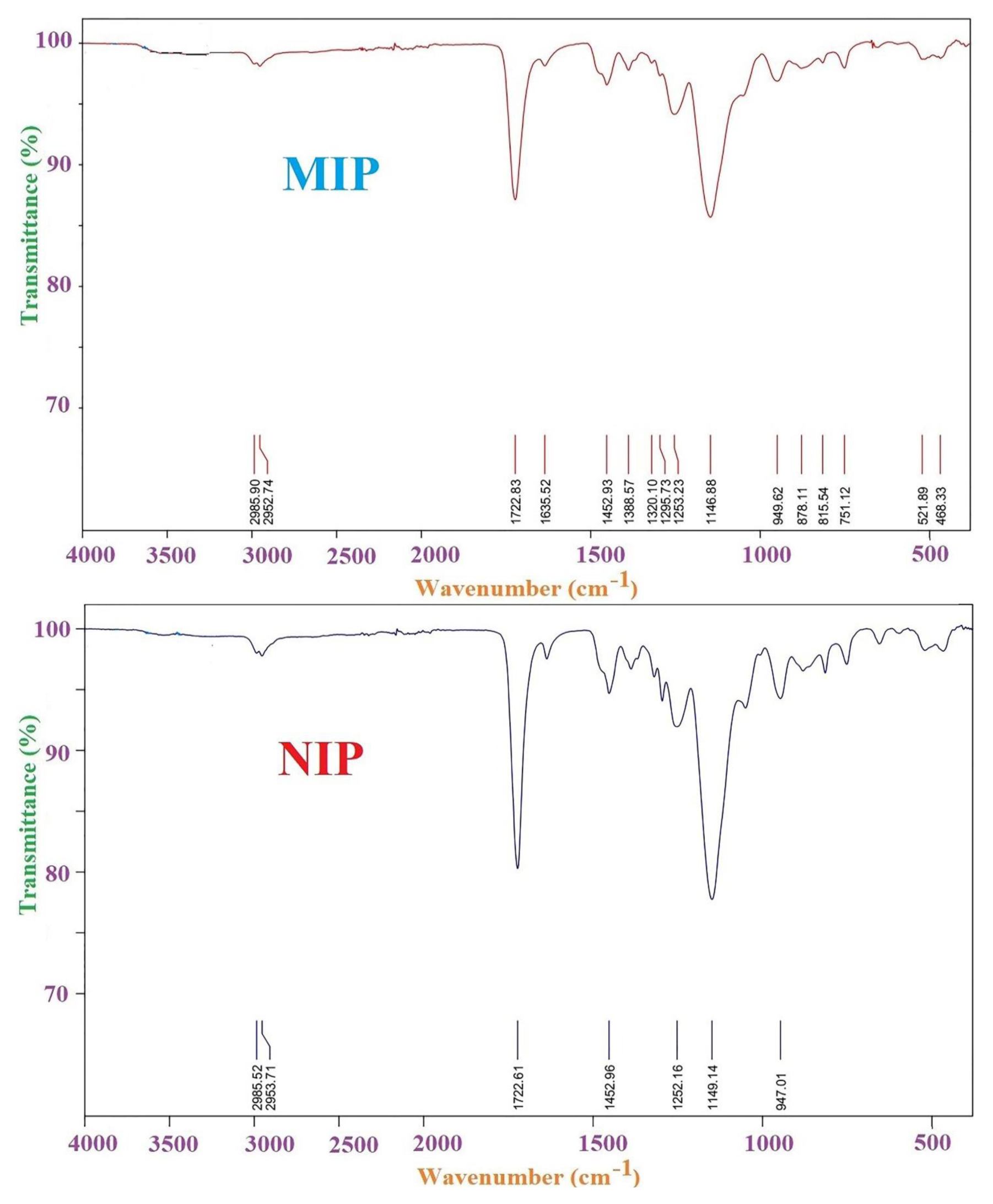

Supplement: Figure S1 — FTIR spectra of MIP and NIP. [file tjc-49-03-267s1.tif]

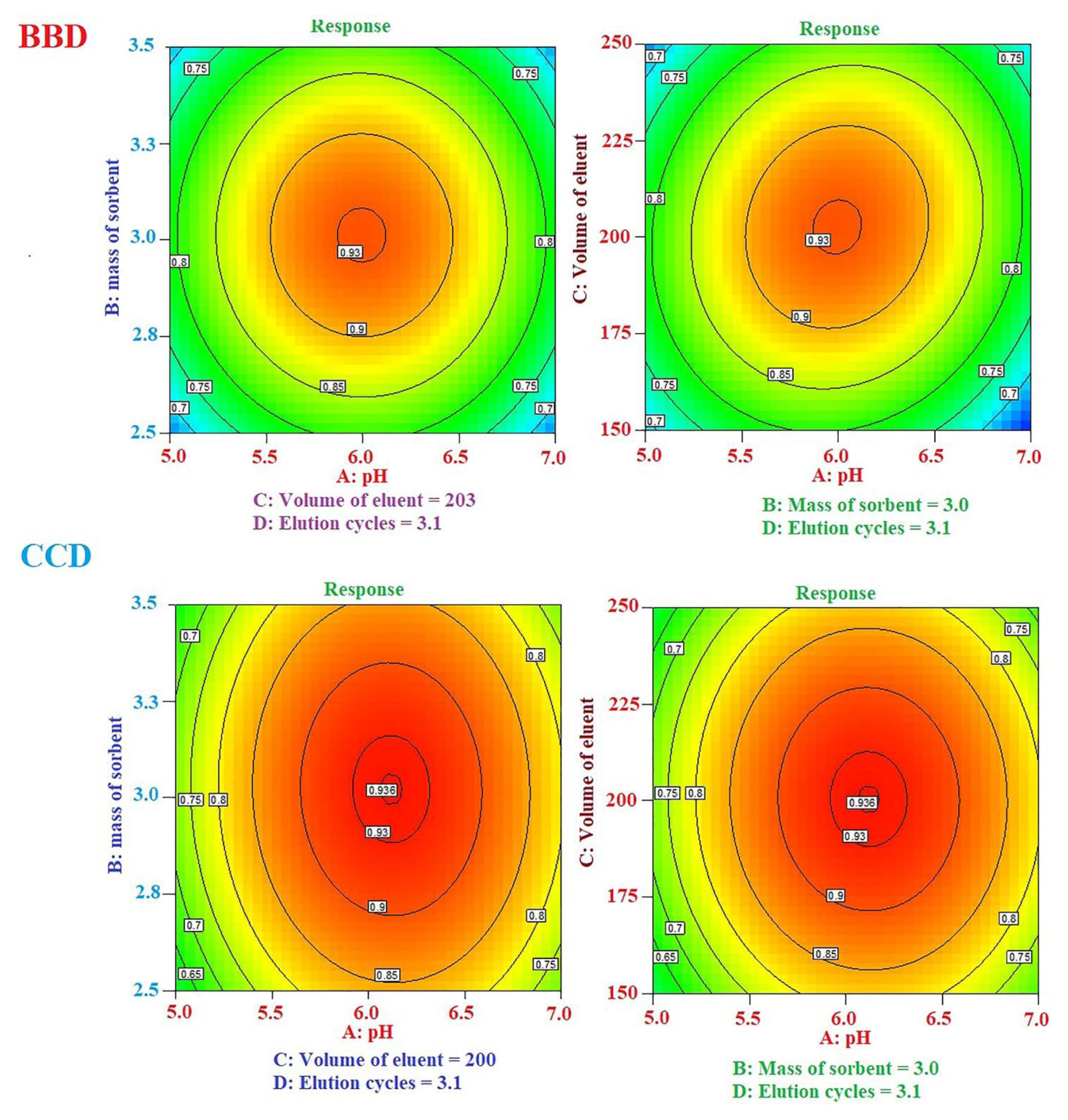

Supplement: Figure S2 — Two-dimensional response surface as the functions of two variables at the center level of other variables. [file tjc-49-03-267s2.tif]
